# 抗PD-1/PD-L1单抗治疗肺癌临床研究进展

**DOI:** 10.3779/j.issn.1009-3419.2015.11.09

**Published:** 2015-11-20

**Authors:** 志煜 黄

**Affiliations:** 310022 杭州，浙江省肿瘤医院胸部肿瘤内科 Department of Thoracic Medical Oncology, Zhejiang Cancer Hospital, Hangzhou 310022, China

**Keywords:** 免疫检验点, PD-1/PD-L1, 单克隆抗体, 肺肿瘤, Checkpoint, PD-1/PD-L1, Monoclonal antibody, Lung neoplasms

## Abstract

近期，抗程序性死亡分子1(programmed death-1, PD-1) /PD-1配体(PD-1 ligand, PD-L1)单抗在晚期非小细胞肺癌的治疗中纷纷取得了突破性进展，相关研究迅速覆盖到肺癌的其他领域，如小细胞肺癌、局部晚期非小细胞肺癌；并且开始尝试与现有治疗手段的各种组合，如联合化疗、靶向药物及放疗等。但是，仍有许多问题有待解决，如寻找理想的预测疗效的生物标记物、探索不同的联合治疗模式、明确原发及继发耐药的机制等等。随着这些问题的相继解决，抗PD-1/PD-L1单抗在肺癌的治疗中将会有更加广阔的应用空间。

## Abstract (en)

Recently, the immune checkpoint inhibitors which target programmed death-1 (PD-1)/PD-1 ligand (PD-L1) have made a breakthrough in the treatment of advanced non-small cell lung cancer. Researches on immune checkpoint inhibitors have been rapidly implemented in various types of lung cancer, such as small cell lung cancer and locally advanced non-small cell lung cancer. These inhibitors have been applied in combination with other treatment strategies, including chemotherapy, targeting therapy and radiotherapy. However, there are still many problems to be solved, such as searching for ideal biomarkers, exploring different combination treatment models, and identifying the potential mechanisms of primary or secondary drug resistance. Along with these problems to be successfully solved, the immune checkpoint inhibitors will have broader applications in lung cancer therapy.

肿瘤的免疫治疗已有200多年历史，但一直未能成为主要的治疗手段；近几年，针对免疫检验点(checkpoint)的单抗治疗不断在癌症治疗中取得突破性进展，尤其在黑色素瘤、肺癌、肾癌及膀胱癌等多种肿瘤的治疗中显示出很好的疗效，初步实现了利用免疫方法治疗肿瘤的梦想。其作用机理简述如下。一般情况下，T细胞的活化需要两个信号^[1]^，T细胞的免疫应答受到复杂的抑制信号(又称为“免疫检验点”)的调节，从而防止发生不可控制的免疫反应甚至是自身免疫性疾病。细胞毒T淋巴细胞相关抗原4(cytotoxic T lymphocyte-associated antigen-4, CTLA-4)和程序性死亡分子1(programmed death-1, PD-1)表达在T细胞表面，同属于抑制性共刺激分子，在免疫系统中扮演着类似“刹车”的角色^[2]^。CTLA-4可与T细胞表面的活化共刺激分子CD28竞争结合配体CD80和CD86，在T细胞活化的早期发挥抑制作用(24 h-48 h内)。因CTLA的配体(即CD80和CD86)只表达在抗原递呈细胞上，而非肿瘤细胞表面，因此CTLA-4抑制T细胞活化发生在次级免疫器官(淋巴结)内，而不是肿瘤微环境中。同时CTLA-4主要表达在CD4+ T细胞而非CD8+ T细胞，CTLA-4单抗的抗肿瘤作用可能是通过增强CD4+ T细胞间接促进CD8+ T细胞的功能^[3, 4]^。PD-1作为另一个关键的抑制性共刺激分子，其表达滞后于CTLA-4。PD-1的配体包括PD-1配体(PD-1 ligand, PD-L1)和PD-L2。PD-L1主要在免疫细胞(如肿瘤浸润淋巴细胞)和上皮细胞(如肿瘤细胞)上诱导性表达(如细胞因子干扰素γ的诱导)，而PD-L2只在APC细胞上表达。因此，不同于CTLA-4的是，PD-1的配体PD-L1在肿瘤细胞及肿瘤浸润淋巴细胞上均有表达，而不是在抗原递呈细胞上，因此PD-1/PD-L1抑制T细胞活化主要在肿瘤微环境中^[5-7]^。目前，在肺癌领域研究得比较多的免疫检验点抑制剂有抗PD-1(nivolumab, pembrolizumab)和PD-L1单抗(MPDL3280A和MEDI-4736)，这些药物已经在肺癌的某些领域取得成功，相关的临床及基础研究仍在如火如荼进行中。现将这方面研究情况综述如下。

## 1 抗PD-1单抗的疗效

抗PD-1单抗主要阻断PD-1受体与它的配体PD-L1(B7-H1)和PD-L2(B7-DC)相结合，从而解除T细胞活性受抑制的状态，促进活化T细胞对肿瘤细胞的攻击。抗PD-1单抗的代表性药物有Nivolumab和Pembrolizumab(MK-3475)。

Nivolumab是一种抗PD-1受体的人源化IgG4型单克隆抗体。Ⅰ期剂量范围扩展的代列研究评估了Nivolumab在296例晚期实体瘤患者中的疗效和安全性，其中非小细胞肺癌(non-small cell lung cancer, NSCLC)患者129例，54%的患者已接受过≥3线的抗肿瘤治疗，患者随机接受Nivolumab 1 mg/kg、3 mg/kg、10 mg/kg，三个剂量等级中的一个，每2周1次^[8]^。122例NSCLC患者可评价疗效，缓解率(overall response rate, ORR)为17.1%，1年、2年和3年生存率分别为42%、24%、18%，总生存期(overall survival, OS)9.9个月^[9]^。进一步分析显示，在所有患者中，3 mg/kg剂量组ORR最高，达32%，OS最长，为14.9个月，因此3 mg/kg被选为后续研究的标准剂量。鳞癌与非鳞癌患者的ORR分别为33%和12%，而两组OS无明显差异。在该研究中，42例患者用免疫组化方法检测了治疗前样本中肿瘤细胞PD-L1的表达，PD-L1表达阳性患者的ORR明显高于阴性者(36% *vs* 0%, *P*=0.006)，初步提示肿瘤细胞PD-L1的表达可能与ORR存在相关性^[16]^。接下来的全球多中心、单臂Ⅱ期临床研究(CheckMate 063)评价了nivolumab治疗晚期、经治的肺鳞癌患者，共纳入117例至少接受过二线化疗后进展的晚期患者，患者ORR为14.5%，中位至起效时间为3.3个月，中位疗效维持时间为6.0个月^[10]^。在2015年美国临床肿瘤协会(American Society of Clinical Oncology, ASCO)会议上同时报告了多项nivolumab治疗肺癌的临床研究。针对鳞癌的Ⅲ期随机对照研究(Checkmate-017研究)共入组272例患者，在二、三线治疗中比较nivolumab与多西他赛，nivolumab组的OS明显好于化疗组(9.2个月 *vs* 6.5个月，HR=0.59，*P*＜0.001)，无进展生存时间(progression-free survival, PFS)(3.5个月 *vs* 2.8个月，HR=0.62，*P*＜0.001)和ORR(20% *vs* 9%，*P*=0.008, 3)均有明显提高，但PD-L1的表达与疗效及预后均无明显相关性^[11]^。Checkmate-057研究则在582例含铂双药方案化疗失败的非鳞型NSCLC中进行，同样观察到nivolumab组的OS及ORR均好于多西他赛；与Checkmate-017研究大相径庭的是，在该研究中，PD-L1的表达能明显预测nivolumab的疗效，即使PD-L1的阳性表达在最低水平时(≥1%)^[12]^。日本学者报道了nivolumab治疗亚裔患者的Ⅱ期研究数据，111例晚期复发NSCLC患者中，鳞癌和非鳞癌患者的ORR分别为25.7%和19.7%^[13]^，其疗效与欧美国家相似。同时报告的还有nivolumab一线治疗晚期NSCLC的Ⅰ期研究，入组52例鳞型及非鳞型NSCLC，ORR 21%，中位OS 98.3周，PD-L1表达阳性患者的ORR高于阴性者(31% *vs* 10%)，但两组OS未见差异^[14]^。在小细胞肺癌(small cell lung cancer, SCLC)的Ⅰ期/Ⅱ期研究中(CheckMate 032)，nivolumab联合或不联合ipilimumab(一种抗CTLA-4抗体)治疗复发SCLC 86例，联合组与单药组的ORR分别为18.0%和17.4%，PD-L1的表达与疗效无关^[15]^。至此，nivolumab在经治晚期NSCLC(包括鳞癌及非鳞癌)中的疗效均得到充分肯定；在晚期NSCLC的一线治疗及复发SCLC的挽救治疗中初步看到了疗效。美国FDA已于2015年3月批准nivolumab用于经治的转移性或晚期鳞型NSCLC。进一步的研究及探索仍在继续，如用于一线治疗，与化疗比较能否胜出？Nivolumab能否与其他治疗手段及治疗药物联合应用？PD-L1的表达能否预测疗效？如何联合两种免疫检验点抑制剂等等。

Pembrolizumab(MK-3475)，是另一种人源化IgG4-κ型单克隆抗体，高选择性阻断PD-1，其作用机理类似Nivolumab。Ⅰ期研究(KEYNOTE-001)入选了495例局部晚期/晚期的NSCLC患者，初治101例患者，经治患者394例；Pembrolizumab的治疗方案为2 mg/kg或10 mg/kg，每3周，或10 mg/kg，每2周，分为研究组182例，验证组313例，用免疫组化方法检测肿瘤细胞PD-L1的表达；结果显示ORR 19.4%，初治及复治患者的ORR分别为24.8%和18.0%，mPFS 3.7个月，mOS 12.0个月^[16]^。PD-L1表达阳性患者的ORR 45.2%，mPFS 6.3个月，中位生存时间未达到，均明显好于PD-L1表达阴性患者；结论认为pembrolizumab在晚期NSCLC表现出明显的抗肿瘤活性，PD-L1(+)能预测疗效^[17]^。在2015年的ASCO会议上还报道了pPembrolizumab的一系列探索性研究。Pembrolizumab联合含铂两药方案一线治疗Ⅲb期/Ⅳ期NSCLC(KEYNOTE-021 CohortA和C组)，总共入组44例患者，Pembrolizumab分别联合泰素+卡铂及培美曲塞+卡铂，ORR分别为30%和58%^[18]^。在KEYNOTE-021 CohortD组研究，初步评估了两种免疫靶向药物的联合，Pembrolizumab联合Ipilimumab治疗晚期复发NSCLC，11例可评价疗效的患者中，1例完全缓解(complete response, CR)(9%)，5例(45%)PR，所有患者疾病稳定≥6周，初步显示联合治疗有很强的抗肿瘤活性，毒性可耐受^[19]^。一项小样本研究针对无症状、未经治疗NSCLC脑转移患者，用Pembrolizumab治疗10例患者，颅脑病灶的ORR达44%^[20]^。Ott等^[21]^报道了KEYNOTE-028研究的初步结果，入选化疗失败的PD-L1(+)的广泛期SCLC患者，目前有20例患者接受治疗，ORR 35%，有效者疗效持续时间已超过16周。这些小样本研究为Pembrolizumab治疗适应症的拓展打下了良好的基础。目前正在开展的大型临床研究有：Ⅱ期/Ⅲ期临床研究(KEYNOTE-010)随机比较Pembrolizumab与多西他塞二线治疗晚期NSCLC；Ⅲ期随机对照研究(KEYNOTE-024, KEYNOTE-042)比较Pembrolizumab与含铂双药化疗一线治疗PD-L1阳性的转移性/晚期NSCLC患者。

## 2 抗PD-L1单抗的疗效

MPDL3280A是人源化抗PDL-1的IgG4型抗体。Ⅰ期研究入选277例不能治愈的各种晚期肿瘤患者接受MPDL3280A单药治疗，全组有175例患者可评价疗效，其中，NSCLC 53例，有效率23%，mPFS 15周，吸烟亚组患者的有效率明显高于非吸烟者(42% *vs* 10%)。检测治疗前样本中肿瘤浸润淋巴细胞(tumor infiltrating lymphocytes, TILs)的PD-L1表达，结果显示，在NSCLC患者中，PD-L1表达3+患者与阴性患者的ORR分别为83%和20%(*P*=0.015)^[22]^。2015年ASCO会议上报道了一项Ⅱ期随机对照研究，比较MPDL3280A与多西他赛二、三线治疗晚期NSCLC(POPLAR研究)，287例患者入组，与化疗相比，在肿瘤细胞或TILs的PD-L1阳性表达者，MPDL3280A明显改善患者的ORR、PFS及OS，TILs及肿瘤细胞的PD-L1阳性表达者更能从治疗中获益^[23]^。另一项Ⅱ期研究用MPDL3280A治疗PD-L1(+)的Ⅲb期/Ⅳ期NSCLC(FIR研究)，分为3个治疗组，第1组为初治患者，第2组为二线及以上的无脑转移患者，第3组为二线及以上的合并无症状脑转移患者，共入组138例患者，114例可评价疗效，初治及经治患者均有良好疗效，PD-L1(3+)患者有更高的ORR^[24]^。Horn等^[25]^报道的Ia期研究，入选88例复发晚期NSCLC患者，同样证明PD-L1(3+)患者的ORR明显高于其他患者(45% *vs* 14%)。从MPDL3280A的相关研究不难发现，PD-L1阳性表达是预测缓解率的良好指标，高表达患者(3+)的有效率可高达45%-83%^[23-25]^。MPDL3280A的相关后续研究基本定位在PD-L1阳性表达患者，目前有多项Ⅱ期、Ⅲ期临床试验仍在进行中。

MEDI-4736是另一个IgG1-k型抗PD-L1单抗。2014年ASCO会议上报道了其治疗多种肿瘤的Ⅰ期临床研究，共有346例晚期肿瘤患者参加了扩展性临床研究，其中NSCLC患者143例，在多种瘤种观察到疗效^[26]^。2015年ASCO会议报道了MEDI-4736治疗经治晚期NSCLC患者198例，非鳞癌116例，鳞癌82例，MEDI-4736剂量10 mg/kg，每2周1次，149例可评价疗效，ORR 14%，疗效持久，PD-L1阳性患者ORR 23%，鳞癌患者的ORR高于非鳞癌(21% *vs* 10%)^[27]^。其他的一些抗PD-L1单抗，如Avelumab(MSB0010718C)亦在NSCLC的治疗中崭露头角^[28]^。这些研究结果提示抗PD-L1单抗治疗晚期NSCLC疗效明显，耐受性良好，PD-L1(+)患者似乎有更好的治疗效果。

综上所述，在晚期、经治NSCLC，无论是抗PD-1单抗还是抗PD-L1单抗均表现出不错的疗效；与标准二线治疗多西他赛相比，在不良反应及疗效上均体现了优越性；各种单抗之间的疗效差距不明显(表 1)；主要的差异体现在不同单抗对PD-L1表达的反应性不一，需要进一步深入研究。

**1:** 抗PD-1/PD-L1单抗的疗效汇总 Clinical Trials on PD-1/PD-L1 monoclonal antibodies in NSCLC

Antibody	Clinical trial	Pathological type	Treatment	*n*	ORR (%)	PFS (m)	OS (m)
PD-1							
Nivolumab	Stage Ⅰ^[9]^	Unclassified	Retreatment	129	17.1	NR	9.9
	Stage Ⅱ^[10]^	SCC	Retreatment	117	14.5	1.9	8.2
	Stage Ⅲ^[11]^	SCC	Retreatment	135	20.0	3.5	9.2
	Stage Ⅲ^[12]^	Non-SCC	Retreatment	292	19.0	2.3	12.2
Pembrolizumab	Stage Ⅰ^[16]^	Unclassified	Primary treatmeny/retreatment	495	19.4	3.7	12.0
PD-L1							
MPDL3280A	Stage Ⅰ^[22]^	Unclassified	Retreatment	53	23.0	15W	NR
	Stage Ⅱ^[23]^	Unclassified	Retreatment	144	15.0	2.8	11.4
MEDI-4736	Stage Ⅰ^[27]^	SCC Non-SCC	Retreatment	82 116	21.0 10.0	NR NR	NR NR
NSCLC: non-small cell lung cancer; ORR: objective response rate; PFS: progression-free survival; OS: overall survival; SCC: squamouse cell carcinama; NR: not reported.							

## 3 抗PD-1/PD-L1单抗的不良反应

Nivolumab的Ⅰ期代列研究显示全组患者药物相关的不良事件(adverse events, AEs)总体发生率为41%，3级/4级药物相关的AEs发生率为14%，主要包括：胃肠道反应、肺炎、皮肤反应等，其中3级/4级肺炎发生率为1%，有3例患者死于肺毒性(2例NSCLC，1例结肠癌)^[8]^。长期安全性观察显示，肺癌组有3例(2%)患者出现治疗相关死亡，均为药物相关性肺炎^[9]^。在Ⅱ期研究中观察到17%患者发生3级/4级治疗相关的AEs，包括乏力、肺炎和腹泻；2例治疗相关死亡，其中1例死于肺炎^[10]^。让人欣慰的是从大样本的Ⅲ期随机对照研究(Checkmate-017研究)中观察到nivolumab安全性良好，与药物相关的3度/4度AEs发生率仅为7%，无治疗相关死亡^[11]^。从CheckMate 032研究中观察到，如果将Nivolumab与Ipilimumab联合，可能会有更高的AEs发生率^[15]^。同为抗PD-1单抗的Pembrolizumab(MK-3475)，其不良反应谱及发生率与Nivolumab类似，≥3度的AEs发生率为9.5%，其中肺炎发生率1.8%，1例肺炎(0.2%)患者死亡^[16]^。抗PD-L1单抗MPDL3280A在Ⅰ期研究中显示有13%的患者出现3级/4级治疗相关的AEs，主要是疲劳、腹泻、高血糖、缺氧，肝酶升高等，未观察到3级-5级的肺炎^[22]^。MEDI-4736的Ⅰ期研究中，≥3度的AEs发生率仅为6%^[27]^。相比抗PD-1单抗，抗PD-L1单抗的耐受性似乎更好，这与其保留了PD-L2通路的活性有一定相关性。纵观抗PD-1/PD-L1单抗的不良反应，与单药的标准二线化疗多西他赛相比^[11, 12]^，总体不良反应发生率明显下降；但不良反应发生谱差异大，前者基本与免疫激活相关，所以在临床应用中需要仔细观察，全程管理。

## 4 寻找预测疗效的生物标记物

PD-1及PD-L1抑制剂治疗晚期NSCLC的有效率大约在20%左右，仍有80%左右的患者对治疗耐受，因此，需要寻找可以预测疗效的生物标记物。PD-L1可在多种肿瘤中表达，据报道大约有27%-50%的肺癌患者表达PD-L1^[29-30]^，也是目前研究得最多的一种生物标记物。PD-L1是个潜在的理想的预测因子吗？从现有的研究结果来看，情形颇为复杂。首先从机理上看，PD-L1的表达并不局限于癌细胞，也可表达在肿瘤相关的巨噬细胞、树突状细胞、成纤维细胞和活化的T细胞中^[31]^；而且PD-L1的表达在整个疾病过程中会有波动，可随时间变化，也可受既往治疗及其他因素诱导，是一种动态的生物标记物；第二：不少研究显示PD-L1(+)可预测PD-1及PD-L1类药物的近期疗效^[8, 17, 22-25, 27]^，但仅有少数研究结果显示能预测生存获益^[17, 22]^；第三，PD-L1表达(-)患者对这类药物也有效^[10, 12]^；第四，PD-L1的预测价值在各种PD-1/PD-L1类抑制剂中并不一致^[8, 10, 20-23, 25]^；第五，从检测的角度来分析，目前有关PD-L1的检测仍存在不少问题。首先各个临床研究在检测PD-L1的表达时，所采用的方法及抗体并不统一；其次选用的观察对象亦有差异，有的研究仅观察肿瘤细胞的PD-L1表达^[16, 17]^，有的研究还检测肿瘤浸润淋巴细胞的PD-L1表达^[22, 23]^；最后，各个研究PD-L1阳性定义的界值(cut-off值)不统一。综上所述，不难发现目前对PD-L1表达状态的检测还不成熟，几种不同的检测技术和分析方法仍在发展中；所以，迫切需要建立标准化的PD-L1检测技术及判读标准。未来研究方向是寻找其他潜在的预测疗效的生物标记物，如基因错配修复状态、肿瘤样本中CD8+的T细胞表达和细胞因子等^[32]^。有学者提出在肿瘤微环境中存在三种免疫细胞浸润情况，不同情况采用PD-1/PD-L1单抗治疗可能会产生不同的结果：很少甚至没有免疫细胞浸润；有免疫细胞浸润但PD-L1的表达量较低；有免疫细胞浸润但被肿瘤细胞通过各种机制诱导无能(包括PD-L1的表达等)^[22]^。总而言之，单一的PD-L1的表达不能准确评估动态的免疫微环境，也无法根据PD-L1的cut-off值捕获所有可能有效的患者，因此很难寻找到理想的预测疗效的生物标记物。尽管如此，通过PD-L1的检测还是能够筛选出更为有效的患者，所以，PD-L1仍然是现阶段PD-1/PD–L1类药物最有前景的预测疗效的生物标记物之一。

## 5 哪个靶点的药物更好？

当前免疫靶向治疗的主要靶点有CTLA-4、PD-1和PD-L1，CTLA-4和PD-1在生物学功能上皆为抑制性受体, 前者表达的部位是参与抗原初次反应的T细胞(激活的CD8+ T细胞)，后者为活化的T细胞、B细胞和NK细胞以及不同类型的肿瘤浸润性淋巴细胞。在黑色素瘤相关研究中发现，抗PD-1抗体的疗效要好于抗CTLA-4抗体^[33]^。在NSCLC，CTLA-4抗体单药治疗鲜有疗效，而PD-1/PD-L1阻断剂的单药均表现出肿瘤活性。

PD-1有两个配体，PD-L1和PD-L2；PD-L1有两个受体PD-1和CD80(B7.1)。所以，尽管抗PD-1抗体和抗PD-L1抗体都作用于PD-1/PD-L1信号轴，但阻断PD-1并不等同于阻断PD-L1。抗PD-1抗体能阻断PD-1与PD-L1、PD-L2结合，却不能阻断PD-L1与CD80相互作用，而抗PD-L1抗体能阻断PD-L1与PD-1、CD80结合，却不能阻断PD-1与PD-L2的结合^[34]^。目前从临床研究中观察到，PD-L1抗体似乎在不良反应上更占优势。临床前模型中已证实PD-1抗体与抗PD-L1抗体的联合应用比单用有更好的疗效^[35]^。因此，PD-1抗体和抗PD-L1抗体在临床疗效和毒性上是否存在差异，两者的联合是否具有现实意义均有待临床试验证实。

## 6 最合适的治疗人群

Nivolumab目前的治疗适应症是鳞型NSCLC。有趣的是，一些研究还观察到吸烟是影响PD-1/PD-L1抗体疗效的一个因素^[22, 36]^。绝大多数鳞型NSCLC都有长期的吸烟史，是否吸烟导致的肺癌对免疫治疗反应更好？如何解释这种现象？目前有研究认为吸烟患者基因突变多且复杂，而基因突变数目越多，免疫细胞识别肿瘤细胞的可能性越大，免疫治疗效果可能更好^[37-39]^。但这种理论并不能解释所有现象，如一些免疫检验点抑制剂在不同病理亚型并未见疗效差异；免疫检验点抑制剂只对少部分患者有效，即使是吸烟的鳞癌患者。因此，如何从病理亚型、分子标记物、临床及基因特征选择最合适的治疗人群值得进一步探索。

## 7 驱动基因与PD-L1之间的相关性

肺腺癌约64%存在驱动基因^[40]^。有关*EGFR*突变与PD-L1表达的相关性，不同的研究得出不一致的结果。Akbay等^[41]^报道在EGFR驱动的小鼠肺癌和人类NSCLC细胞系模型，EGFR信号通路活化和PD-L1、PD-1和CTLA-4的上调相关。相应地，多个研究者^[42-44]^报告EGFR活性突变患者存在PD-L1的高表达。与此相反，也有研究^[45]^显示PD-L1的表达与驱动基因的表达无明显关联。还有研究显示在EGFR突变及ALK(+)患者，PD-L1表达高低在治疗前后会有动态变化^[46]^。进一步的基础研究报道，EGFR可能通过p-ERK1/2/p-c-Jun通路活化上调PD-L1表达，EGFR TKI不仅能直接抑制肿瘤细胞的活性，还能通过下调PD-L1的表达间接增强机体的抗肿瘤免疫，故研究者认为PD-1/PD-L1抗体可能是EGFR-TKI敏感突变患者，尤其是获得性耐药患者的治疗方向^[47]^。同时，也有研究^[43]^探索*K*-*RAS*等突变基因与PD-L1表达之间的关联性。最近有研究^[48]^报道肺癌的基因组景观将影响抗PD-1治疗的疗效。因此，如何将靶向药物与免疫检验点抑制剂进行有机结合将是非常有意义的研究领域。

## 8 展望未来

虽然目前CTLA-4和PD-1/PD-L1的单抗在临床上受到格外关注，但筛选另外新的免疫检验点并进行阻断可能会在临床上表现出更好疗效。一方面很多免疫抑制检验点在肿瘤细胞中与PD-1/PD-L1共表达，另一方面，免疫抑制检验点的联合阻断中可供选择的目标增多有利于筛选出最佳组合从而达到最佳效果。我们将介绍一些已经进入临床试验或者即将进入临床试验的下一代免疫抑制检验点的分子靶点：淋巴细胞活化基因3(LAG-3)是表达在活化T细胞、NK细胞及B细胞上的免疫抑制检验点分子^[49-51]^。目前已知的唯一配体是MHC-Ⅱ分子。动物实验表明LAG-3单抗可以抑制肿瘤微环境中的调节性T细胞(Treg细胞)的活性^[52]^，从而恢复机体抗肿瘤免疫活性。目前已有1个LAG-3单抗进入Ⅰ期临床实验(临床实验：NCT01968109)。NK细胞作为固有免疫细胞，直接可与肿瘤细胞表面的MHC-Ⅰ分子结合发挥杀伤肿瘤细胞的作用。NK细胞表面存在杀伤抑制受体(KIRs，可抑制NK细胞的杀伤作用)，在肿瘤微环境中可能会被诱导表达，从而抑制NK细胞的杀伤功能。因此，KIRs也被认为是免疫抑制检验点，通过阻断其信号可增强NK细胞的杀伤肿瘤的功能^[53]^。目前已有针对KIRs单抗进入临床实验(lirilumab单抗治疗急性粒细胞白血病已进入Ⅰ期临床实验)^[54]^，但在肺癌中尚未有临床实验注册。KIRs单抗联合CTLA-4单抗或PD-1/PD-L1单抗可以联合激活固有免疫和适应性免疫，在免疫治疗中显示出诱人的前景。T细胞免疫球蛋白和粘蛋白-3(TIM-3)是表达在活化的T细胞、NK细胞和单核细胞表面的免疫抑制检验点分子。基础实验显示TIM-3和LAG-3分子有相似的免疫调节功能，同时TIM-3几乎广泛与PD-1共表达于肿瘤浸润淋巴细胞上^[55]^。联合阻断PD-1和TIM-3比单一阻断其中单一分子可更有效的控制肿瘤的生长，显示出较好的临床应用前景。目前LAG-3的单抗正处于研发阶段，尚未进入临床实验。

## 9 小结

免疫检验点抑制剂已经在肺癌的治疗中初露锋芒，Nivolumab抢先在肺鳞癌的二线治疗中争得一席之地，各种类似药物及相关研究正在火热进行中，但是仍有许多问题悬而未决：①寻找理想的预测疗效的生物标记物；②明确原发及继发耐药的机制；③探索不同的治疗模式：用于一线还是二、三线治疗？单药或者联合治疗？联合化疗、放疗还是靶向药物^[56]^？两种免疫检查点抑制剂的联合虽然已经在晚期黑色素瘤的治疗中得到肯定^[57]^，在肺癌患者是否会重复出同样的故事？④存在驱动基因的患者，如何将抗PD-1/PD-L1抗体与靶向治疗进行整合？⑤寻找更有效的免疫检验点抑制剂。随着这些问题的解决，相信免疫检验点抑制剂将会对肺癌的临床实践带来革命性的改变。
